# Association of Daily Step Count and Intensity With Incident Dementia in 78 430 Adults Living in the UK

**DOI:** 10.1001/jamaneurol.2022.2672

**Published:** 2022-09-06

**Authors:** Borja del Pozo Cruz, Matthew Ahmadi, Sharon L. Naismith, Emmanuel Stamatakis

**Affiliations:** 1 Department of Sports Science and Clinical Biomechanics, Centre for Active and Healthy Ageing, University of Southern Denmark, Odense, Denmark; 2 Charles Perkins Centre, Faculty of Medicine and Health, School of Health Sciences, The University of Sydney, Camperdown, New South Wales, Australia; 3 Charles Perkins Centre, Faculty of Science, The University of Sydney, Camperdown, New South Wales, Australia; 4 Charles Perkins Centre, Faculty of Medicine and Health, School of Health Sciences, The University of Sydney, Camperdown, New South Wales, Australia

## Abstract

**Importance:**

Step-based recommendations may be appropriate for dementia-prevention guidelines. However, the association of step count and intensity with dementia incidence is unknown.

**Objective:**

To examine the dose-response association between daily step count and intensity and incidence of all-cause dementia among adults in the UK.

**Design, Setting, and Participants:**

UK Biobank prospective population-based cohort study (February 2013 to December 2015) with 6.9 years of follow-up (data analysis conducted May 2022). A total of 78 430 of 103 684 eligible adults aged 40 to 79 years with valid wrist accelerometer data were included. Registry-based dementia was ascertained through October 2021.

**Exposures:**

Accelerometer-derived daily step count, incidental steps (less than 40 steps per minute), purposeful steps (40 steps per minute or more), and peak 30-minute cadence (ie, mean steps per minute recorded for the 30 highest, not necessarily consecutive, minutes in a day).

**Main Outcomes and Measures:**

Incident dementia (fatal and nonfatal), obtained through linkage with inpatient hospitalization or primary care records or recorded as the underlying or contributory cause of death in death registers. Spline Cox regressions were used to assess dose-response associations.

**Results:**

The study monitored 78 430 adults (mean [SD] age, 61.1 [7.9] years; 35 040 [44.7%] male and 43 390 [55.3%] female; 881 [1.1%] were Asian, 641 [0.8%] were Black, 427 [0.5%] were of mixed race, 75 852 [96.7%] were White, and 629 [0.8%] were of another, unspecified race) over a median (IQR) follow-up of 6.9 (6.4-7.5) years, 866 of whom developed dementia (mean [SD] age, 68.3 [5.6] years; 480 [55.4%] male and 386 [54.6%] female; 5 [0.6%] Asian, 6 [0.7%] Black, 4 [0.4%] mixed race, 821 [97.6%] White, and 6 [0.7%] other). Analyses revealed nonlinear associations between daily steps. The optimal dose (ie, exposure value at which the maximum risk reduction was observed) was 9826 steps (hazard ratio [HR], 0.49; 95% CI, 0.39-0.62) and the minimal dose (ie, exposure value at which the risk reduction was 50% of the observed maximum risk reduction) was 3826 steps (HR, 0.75; 95% CI, 0.67-0.83). The incidental cadence optimal dose was 3677 steps (HR, 0.58; 95% CI, 0.44-0.72); purposeful cadence optimal dose was 6315 steps (HR, 0.43; 95% CI, 0.32-0.58); and peak 30-minute cadence optimal dose was 112 steps per minute (HR, 0.38; 95% CI, 0.24-0.60).

**Conclusions and Relevance:**

In this cohort study, a higher number of steps was associated with lower risk of all-cause dementia. The findings suggest that a dose of just under 10 000 steps per day may be optimally associated with a lower risk of dementia. Steps performed at higher intensity resulted in stronger associations.

## Key Points

**Question:**

Is there a dose-response association of daily step count and intensity with incidence of all-cause dementia among adults living in the UK?

**Findings:**

This cohort study of adults assessed with wrist-worn accelerometers found that accruing more steps per day was associated with steady declines in dementia incidence risk, up to 9800 steps per day, beyond which the benefits upturned. The dose associated with 50% of maximal observed benefit was 3800 steps per day, and steps at higher intensity (cadence) were associated with lower incidence risk.

**Meaning:**

The findings in this study suggest that accumulating more steps per day just under the popular threshold of 10 000 steps per day and performing steps at higher intensity may be associated with lower risk of dementia onset.

## Teaser

This cohort study evaluates the association between all-cause dementia and daily step count and intensity in adults using UK Biobank data.

## Introduction

Step count is a popular approach to providing physical activity targets for the general public.^1^ An optimal dose of 6000 to 8000 steps has been suggested to reduce the risk of all-cause mortality.^2^ Higher step counts may lower the risk of cardiovascular and cancer mortality^3^ and incident diabetes, particularly more intense steps.^4^ Step-based physical activity targets are easy to grasp and memorize and may be ideal for dementia-prevention guidelines.^5,6^ To our knowledge, no study on the dose-response association of daily steps and stepping intensity (ie, cadence or steps per minute) with incident dementia exists. Understanding this association is critical to determining the optimal dose of stepping volume and intensity for dementia prevention. We examined the dose-response association of daily step count and intensity with incident all-cause dementia in a large population sample of adults in the UK who wore wrist accelerometers.

## Methods

**Figure 1.**
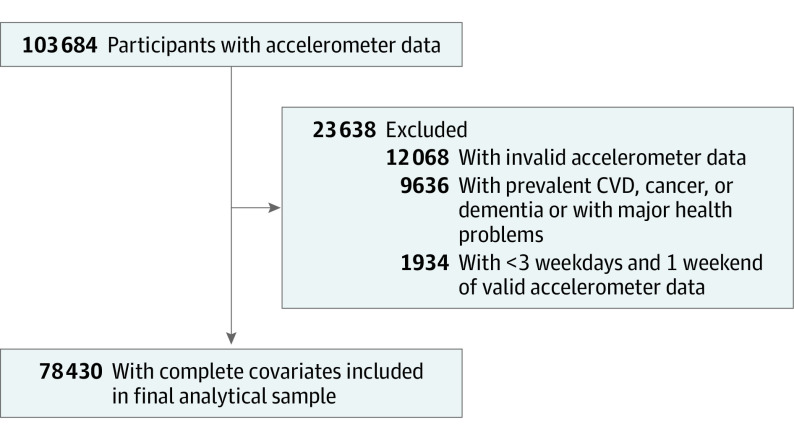
Flow Diagram of Study Participants CVD indicates cardiovascular disease.

This study used data from UK Biobank (February 2013 to December 2015)^7^ and followed the Strengthening the Reporting of Observational Studies in Epidemiology (STROBE) reporting guideline. All participants provided written informed consent. The study was approved by the National Health Service and the National Research Ethics Service (reference 11/NW/0382). There were 236 519 eligible participants who provided a valid email address. Of these, 103 684 accepted the invitation and were instructed to wear an Axivity AX3 accelerometer on their dominant wrist 24 hours a day, 7 days a week, to measure physical activity. A total of 78 430 participants aged 40 to 79 years with at least 3 valid days (more than 16 hours wearing time) and complete data on covariates, and who were free of cardiovascular disease, cancer, or dementia at baseline were included in the analysis (Figure 1). Participants were monitored through October 31, 2021, with incident dementia (fatal and nonfatal) obtained through linkage with inpatient hospitalization or primary care records, or recorded as the underlying or contributory cause of death in the death registers.^8^ We identified walking activities using an accelerometer-based activity machine learning scheme^9^ and used a validated step-count algorithm^10^ for wrist accelerometers to estimate the number of steps. We used cadence-based stepping metrics reflective of pace and intensity under free-living conditions: incidental steps, defined as fewer than 40 steps per minute (eg, indoor walking from one room to another);^11^ purposeful steps, defined as 40 or more steps per minute (eg, steps while exercising);^11^ and peak 30-minute cadence (ie, average steps per minute recorded for the 30 highest, not necessarily consecutive, minutes in a day).^12^

The sample was described by tertiles of daily steps count using means (SDs) and percentages for continuous and categorical variables, respectively. We assessed the dose-response association between step-based metrics and incident all-cause dementia using restricted cubic splines. Knots were placed at the 10th, 50th, and 90th percentiles of the exposure distribution. Nonlinearity was assessed by a Wald test. We estimated the optimal dose (ie, exposure value at which the maximum significant risk reduction was observed) and the minimal dose (ie, exposure value at which the risk reduction was 50% of the observed maximum significant risk reduction). E-values associated with the optimal dose^13^ were calculated, estimating the plausibility of bias from unmeasured confounding. Models were adjusted for age, sex, race, education, socioeconomical status, smoking, alcohol use, fruit and vegetable consumption, family history of cardiovascular disease and cancer, medication use, accelerometer-measured sleep, and valid accelerometer wear days. Race was included as a potential confounder in the association between steps and incidence of dementia, and data were collected via self-report. Participants were offered multiple choice and the categories were taken from the UK Office for National Statistics. Models for incidental steps were adjusted for purposeful steps and vice versa. For peak 30-minute cadence, models were adjusted for daily steps. To minimize chances of reverse causation, we ran a sensitivity analysis removing participants who were diagnosed with dementia within the first 2 years of follow-up. An additional model was adjusted for cholesterol, hemoglobin A_1c_, body mass index, and mean arterial pressure. We used R version 4.2.1 (R Foundation) in our analyses. Two-tailed *P* values less than .05 were considered significant.

## Results

**Figure 2.**
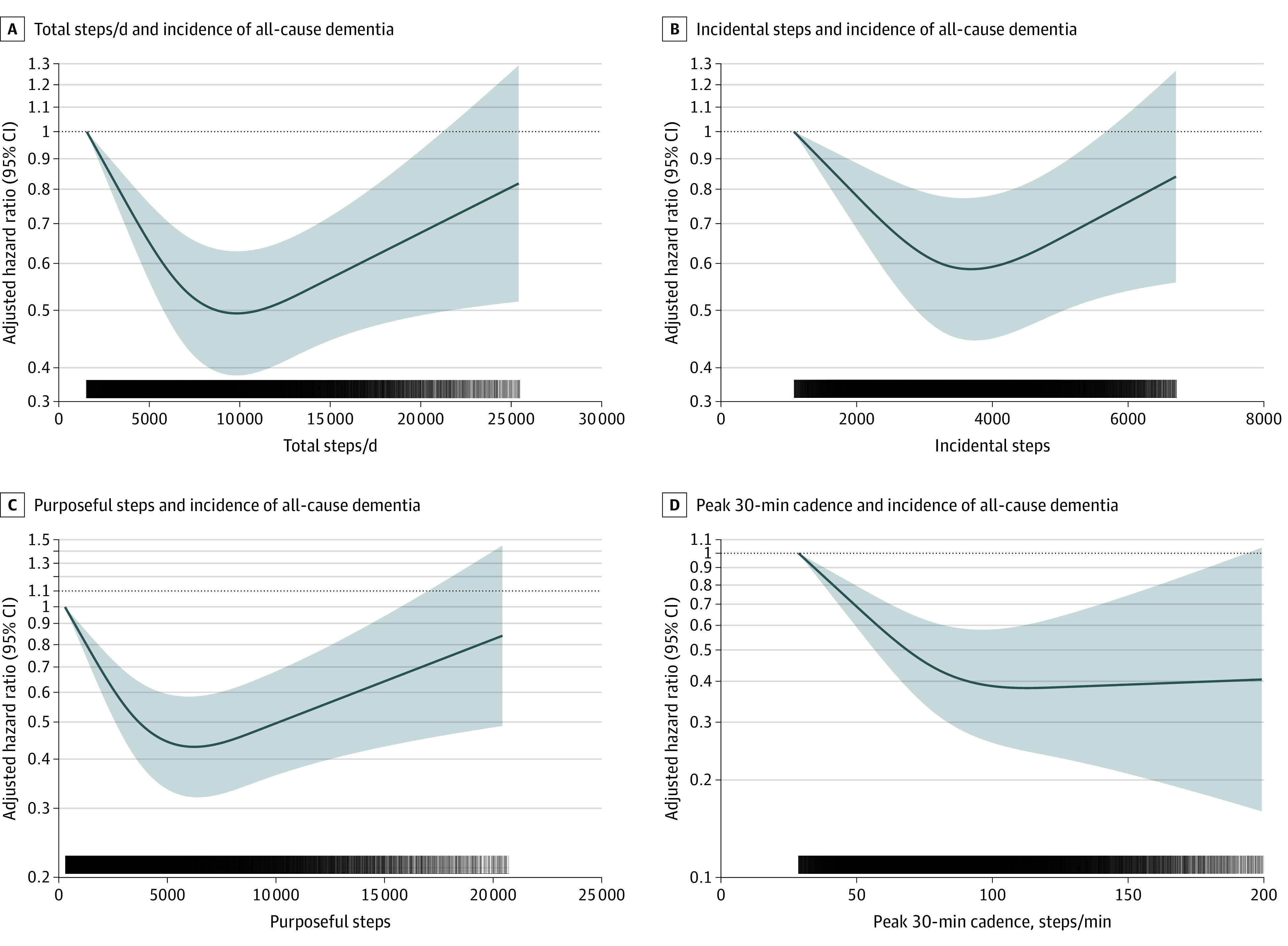
Dose-Response Association Between Different Accelerometer-Measured Step-Based Metrics and Incidence of All-Cause Dementia Shading indicates 95% CIs; solid lines, hazard ratios, in logarithmic scale, adjusted for age, sex, race, education, Townsend deprivation index, smoking, alcohol use, fruit and vegetable consumption, family history of cardiovascular disease and cancer, medication use (cholesterol, insulin, and hypertension), accelerometer-measured sleep, and days wearing accelerometer. For incidental steps, models were further adjusted for purposeful steps (and vice versa). For peak 30-minute steps, models were additionally adjusted for total steps per day. Total steps per day indicates the mean number of steps accumulated in a day; incidental steps, the total daily steps at 1-39 steps per minute; purposeful steps, the total daily steps at ≥40 steps per minute; peak 30-minute cadence, the mean steps per minute recorded for the 30 highest, not necessarily consecutive, minutes in a day. Dose-response associations were assessed with restricted cubic splines with knots at 10th, 50th, and 90th centiles of the distribution of the exposure of interest.

Among the 78 430 participants in this study, the mean (SD) age was 61.1 (7.9) years; 35 040 participants (44.7%) were male and 43 390 (55.3%) were female; 881 participants were [1.1%] were Asian, 641 [0.8%] were Black, 427 [0.5%] were of mixed race, 75 852 [96.7%] were White, and 629 [0.8%] were of another, unspecified race. Over a median (IQR) follow-up of 6.9 (6.4-7.5) years, 866 participants developed dementia (mean [SD] age, 68.3 [5.6] years; 480 [55.4%] male and 386 [54.6%] female; 5 [0.6%] Asian, 6 [0.7%] Black, 4 [0.4%] mixed race, 821 [97.6%] White, and 6 [0.7%] other). Younger, healthier (defined as lower rates of alcohol consumption and tobacco use and higher rates of fruit and vegetable consumption) female participants took more steps in the sample (Table). We found nonlinear associations for daily steps, wherein the optimal dose was 9826 steps (hazard ratio [HR], 0.49; 95% CI, 0.39-0.62) and the minimal dose was 3826 steps (HR, 0.75; 95% CI, 0.67-0.83; E-value, 3.46 [upper CI, 2.55]). For incidental steps, the optimal dose was 3677 steps (HR, 0.58; 95% CI, 0.44-0.72; E-value, 2.80 [upper CI, 1.91]). For purposeful steps, the optimal dose was 6315 steps (HR, 0.43; 95% CI, 0.32-0.58; E-value, 4.07 [upper CI, 2.82]). For peak 30-minute cadence, the optimal dose was 112 steps per minute (HR, 0.38; 95% CI, 0.24-0.60; E-value, 4.65 [upper CI, 2.71]) (Figure 2). Removing participants diagnosed with dementia within the first 2 years of follow-up (eFigure 1 in the Supplement) or further adjustment for relevant biomarkers (eFigure 2 in the Supplement) did not change the results.

**Table.** Baseline Characteristics of Study Participants by Tertiles of Mean Daily Accelerometer-Measured Step Count

Characteristic	Mean (SD)				*P* value^a^
	Overall	Tertile 1 (1540 to <5386 steps)	Tertile 2 (5386 to <8821 steps)	Tertile 3 (≥8821 steps)	
Sample size, No.	78 430	26 149	26 151	26 150	NA
Age, y	61.1 (7.9)	62.9 (7.7)	60.9 (7.8)	59.6 (7.8)	<.001
Female, No. (%)	43 390 (55.3)	14 605 (55.9)	14 225 (54.4)	14 580 (55.8)	.001
Male, No. (%)	35 040 (44.7)	11 544 (44.1)	11 926 (45.6)	11 570 (44.2)	
Race, No. (%)^b^					
Asian	881 (1.1)	336 (1.3)	298 (1.1)	247 (0.9)	.01
Black	641 (0.8)	221 (0.8)	203 (0.8)	217 (0.8)	
Mixed race	427 (0.5)	131 (0.5)	153 (0.6)	143 (0.5)	
White	75 852 (96.7)	25 221 (96.5)	25 294 (96.7)	25 337 (96.9)	
Other^c^	629 (0.8)	232 (0.9)	197 (0.8)	200 (0.8)	
Country of origin, No. (%)					
England	70326 (89.7)	23563 (90.1)	23429 (89.6)	23334 (89.3)	<.001
Scotland	5190 (6.6)	1591 (6.1)	1748 (6.7)	1851 (7.1)	
Wales	2914 (3.7)	987 (3.8)	968 (3.7)	959 (3.7)	
University degree, No. (%)	43 356 (55.3)	14 799 (56.6)	14 253 (54.5)	14 304 (54.7)	<.001
Townsend deprivation index score (lower scores indicate higher affluence)	−1.77 (2.79)	−1.72 (2.82)	−1.80 (2.79)	−1.79 (2.76)	.001
Smoking, never, No. (%)	45 330 (57.8)	14 612 (55.9)	15 213 (58.2)	15 505 (59.3)	<.001
Alcohol use within guidelines,^a^ No. (%)	28 912 (36.9)	9327 (35.7)	9768 (37.4)	9817 (37.5)	<.001
Fruit consumption, servings/d	3.22 (2.49)	3.09 (2.45)	3.19 (2.38)	3.37 (2.62)	<.001
Vegetable consumption, servings/d	4.89 (3.13)	4.81 (3.03)	4.89 (3.21)	4.98 (3.13)	<.001
Family history of CVD, No. (%)	42 885 (54.7)	14 809 (56.7)	14 235 (54.4)	13 841 (52.9)	<.001
Family history of cancer, No. (%)	19 556 (24.9)	6676 (25.5)	6552 (25.1)	6328 (24.2)	.002
Cholesterol medication, No. (%)	10 645 (13.6)	4854 (18.6)	3300 (12.6)	2491 (9.5)	<.001
Insulin medication, No. (%)	470 (0.6)	225 (0.9)	136 (0.5)	109 (0.4)	<.001
Hypertension medication, No. (%)	12 480 (15.9)	5585 (21.4)	3909 (15.0)	2986 (11.4)	<.001
HbA_1c_, % total hemoglobin,^d^ mean (SD)	5.38 (0.49)	5.44 (0.58)	5.36 (0.46)	5.33 (0.41)	<.001
High-density lipoprotein cholesterol, mg/dL^e^	57.53 (14.17)	55.60 (14.17)	57.53 (14.17)	59.46 (15.06)	<.001
Low-density lipoprotein cholesterol, mg/dL^e^	137.84 (32.43)	138.22 (0.88)	138.22 (32.43)	137.45 (31.66)	<.001
Triglycerides, mg/dL^f^	146.02 (84.96)	155.75 (86.73)	146.02 (85.84)	136.28 (80.53)	<.001
Arterial blood pressure, mm Hg	100.56 (12.34)	101.70 (12.41)	100.46 (12.24)	99.49 (12.28)	<.001
Sleep, accelerometer-measured, min/d	421.56 (85.55)	414.12 (96.92)	422.79 (86.99)	427.76 (80.41)	<.001
Accelerometer wear days	6.90 (0.37)	6.89 (0.41)	6.90 (0.37)	6.92 (0.34)	<.001
Total steps/d^g^	8040.59 (4932.97)	3761.76 (1079.93)	6982.20 (977.70)	13 377.38 (4790.68)	<.001
Incidental steps/d^h^	3417.60 (1266.29)	2278.90 (641.90)	3438.17 (758.25)	4535.61 (1129.85)	<.001
Purposeful steps/d^i^	4622.99 (4160.15)	1482.86 (717.76)	3544.03 (994.41)	8841.77 (4646.91)	<.001
Peak 30-min cadence, steps/min^j^	84.40 (34.46)	54.47 (13.80)	81.22 (15.37)	117.51 (33.66)	<.001

Abbreviations: CVD, cardiovascular disease; HbA_1c_, hemoglobin A_1c_; NA, not applicable.

^a^ Guidelines for alcohol use in the UK recommend no more than 14 units of alcohol per week for both men and women.

^b^ Race was included as a potential confounder in the association between steps and incidence of dementia, and data were collected via self-report using multiple choice according to the categories set by the UK Office for National Statistics.

^c^ Included other, unspecified race if presented multiple-choice categories did not apply.

^d^ To convert to mmol/mol, multiply by 10.93 and subtract 23.5.

^e^ To convert to mmol/L, multiply by 0.0259.

^f^ To convert to mmol/L, multiply by 0.0113.

^g^ Mean number of steps accumulated in a day.

^h^ Total daily steps at 1-39 steps/min.

^i^ Total daily steps at ≥40 steps/min.

^j^ Mean steps/min recorded for the 30 highest, not necessarily consecutive, minutes in a day.

## Discussion

We found nonlinear associations of daily steps and intensity with incident dementia. These results may have implications for public health. We found no minimal threshold for the beneficial association of step counts with incident dementia. Our findings suggest that approximately 9800 steps per day may be optimal to lower the risk of dementia. We estimated the minimum dose at approximately 3800 steps per day, which was associated with 25% lower incident dementia. Other studies have found 4400 steps to be associated with mortality outcomes.^3,11^ This finding suggests that population-wide dementia prevention might be improved by shifting away from the least-active end of the step-count distributions. Unlike previous studies investigating mortality outcomes,^3^ our analyses highlight the importance of stepping intensity for preventing dementia. Both purposeful steps and peak 30-minute cadence (ie, an indicator of overall best natural effort in a free-living environment) were associated with lower risks of dementia.^10^

Strengths of this study are the large sample of adults with accelerometers and the use of multisource registry-based prospectively collected data to ascertain incident dementia. This study represents an important contribution to step count–based recommendations for dementia prevention. Step count–based recommendations have the advantage of being easy to communicate, interpret, and measure,^11,14^ and may be particularly relevant for people who accumulate their physical activity in an unstructured manner. For such individuals, it may be otherwise challenging to track physical activity or determine whether they are sufficiently active relative to current minute- and intensity-based physical activity guidelines (ie, 150 to 300 minutes per week of moderate to vigorous physical activity). Therefore, step-based recommendations could provide informative supplementary information to the current physical activity guidelines.

### Limitations

Limitations of this study include its observational design and the low response rate (5.5%) of participants in UK Biobank, although studies^15^ have demonstrated this poor representativeness does not necessarily influence associations between physical activity and health outcomes. Reverse causation and residual confounding may still be present. However, the large E-values showed this possibility is minimal. The inversion of the right part of the dose-response curves in this study likely reflects the sparsity of data and events rather than a genuine lack of beneficial association at higher levels of stepping. The age range of participants may have resulted in limited dementia cases, meaning our results may not be generalizable to older populations. Because there are often considerable delays in dementia diagnosis, and this study did not include formal clinical and cognitive assessments of dementia, it is possible that the prevalence of dementia in the community was much higher.

## Conclusions

Taking more steps per day was associated with a lower risk of incident all-cause dementia. The optimal dose was estimated at 9800 steps per day, just under the popular target of 10 000 steps. Intensity of stepping resulted in stronger associations. Future guidelines for dementia prevention may capitalize on the results of this study to promote step-based recommendations.

## Notes

Supplement. eFigure 1. Dose–response association (Adjusted hazard ratios, solid line and associated 95% confidence interval band, dotted lines) between different accelerometer-measured step-based metrics and incidence of all-cause dementia after excluding dementia diagnoses within the first 2 years of follow-up eFigure 2. Dose–response association (Adjusted hazard ratios, solid line and associated 95% confidence interval band, dotted lines) between different accelerometer-measured step-based metrics and incidence of all-cause dementia with further adjustment for relevant metabolic biomarkers
