# Supplementary material for: Association of Daily Step Count and Intensity With Incident Dementia in 78 430 Adults Living in the UK
Source: JAMA Neurol. 2022 Sep 6;79(10):1059–63. doi: 10.1001/jamaneurol.2022.2672 (PMC9449869; doi:10.1001/jamaneurol.2022.2672)
Supplement: Supplement. — eFigure 1. Dose–response association (Adjusted hazard ratios, solid line and associated 95% confidence interval band, dotted lines) between different accelerometer-measured step-based metrics and incidence of all-cause dementia after excluding dementia diagnoses within the first 2 years of follow-up eFigure 2. Dose–response association (Adjusted hazard ratios, solid line and associated 95% confidence interval band, dotted lines) between different accelerometer-measured step-based metrics and incidence of all-cause dementia with further adjustment for relevant metabolic biomarkers [file jamaneurol-e222672-s001.pdf]

Supplemental Online Content

del Pozo Cruz B, Ahmadi M, Naismith SL, Stamatakis E. Association of daily step count and intensity with incident dementia in 78 430 adults living in the UK. *JAMA Neurol*. Published online September 6, 2022. doi:10.1001/jamaneurol.2022.2672

eFigure 1. Dose–response association (Adjusted hazard ratios, solid line and associated 95% confidence interval band, dotted lines) between different accelerometer-measured step-based metrics and incidence of all-cause dementia after excluding dementia diagnoses within the first 2 years of follow-up

eFigure 2. Dose–response association (Adjusted hazard ratios, solid line and associated 95% confidence interval band, dotted lines) between different accelerometer-measured step-based metrics and incidence of all-cause dementia with further adjustment for relevant metabolic biomarkers

This supplemental material has been provided by the authors to give readers additional information about their work.

© 2022 del Pozo Cruz et al. *JAMA Neurology.*

eFigure 1. Dose–response association (Adjusted[a] hazard ratios, solid line and associated 95% confidence interval band, dotted lines) between different accelerometer-measured step-based metrics and incidence of all-cause dementia after excluding dementia diagnoses within the first 2 years of follow-up

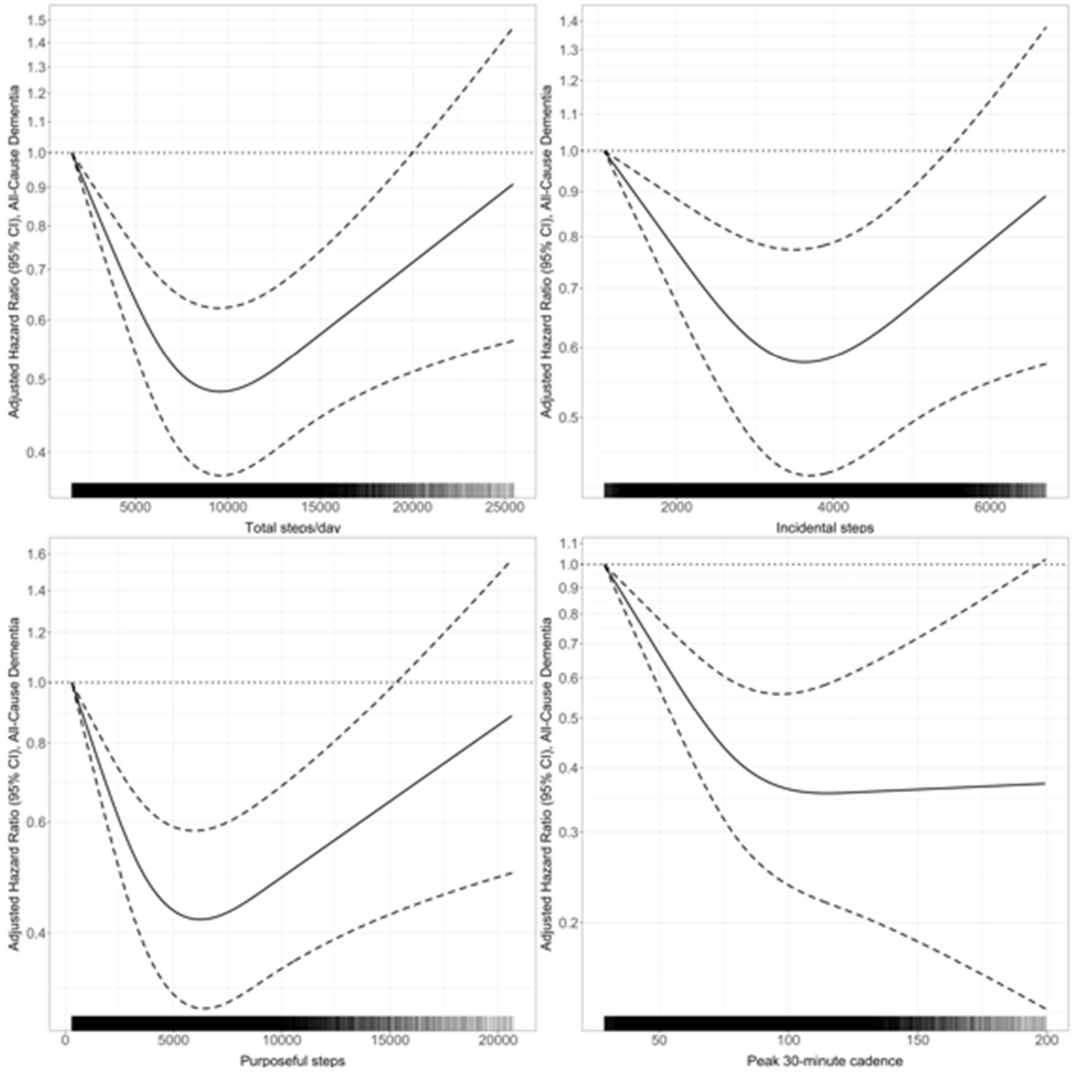

[a]Adjusted for age, sex, ethnicity, education, Townsend deprivation index, smoking, alcohol use, fruit and vegetable consumption, family history of CVD and Cancer, medication use (cholesterol, insulin, and hypertension), accelerometer-measured sleep, and wear accelerometer days. For incidental steps, models were further adjusted for purposeful steps (and vice versa). For peak 30-min steps models were additionally adjusted for total steps per day. Total steps/day, Average of steps accumulated in a day; Incidental steps, Total daily steps at 1-39 steps/minute; Purposeful steps, Total daily steps at ≥40 steps/minute, Peak 30-min cadence, Average steps/minute recorded for the 30 highest, but not necessarily consecutive, minutes in a day. Dose-response associations were assessed with restricted cubic splines with knots at 10th,

© 2022 del Pozo Cruz et al. *JAMA Neurology.*

50th, and 90th centiles of the distribution of the exposure of interest. Hazard ratios are in logarithmic scale.

© 2022 del Pozo Cruz et al. *JAMA Neurology.*

eFigure 2. Dose–response association (Adjusted[a] hazard ratios, solid line and associated 95% confidence interval band, dotted lines) between different accelerometer-measured step-based metrics and incidence of all-cause dementia with further adjustment for relevant metabolic biomarkers

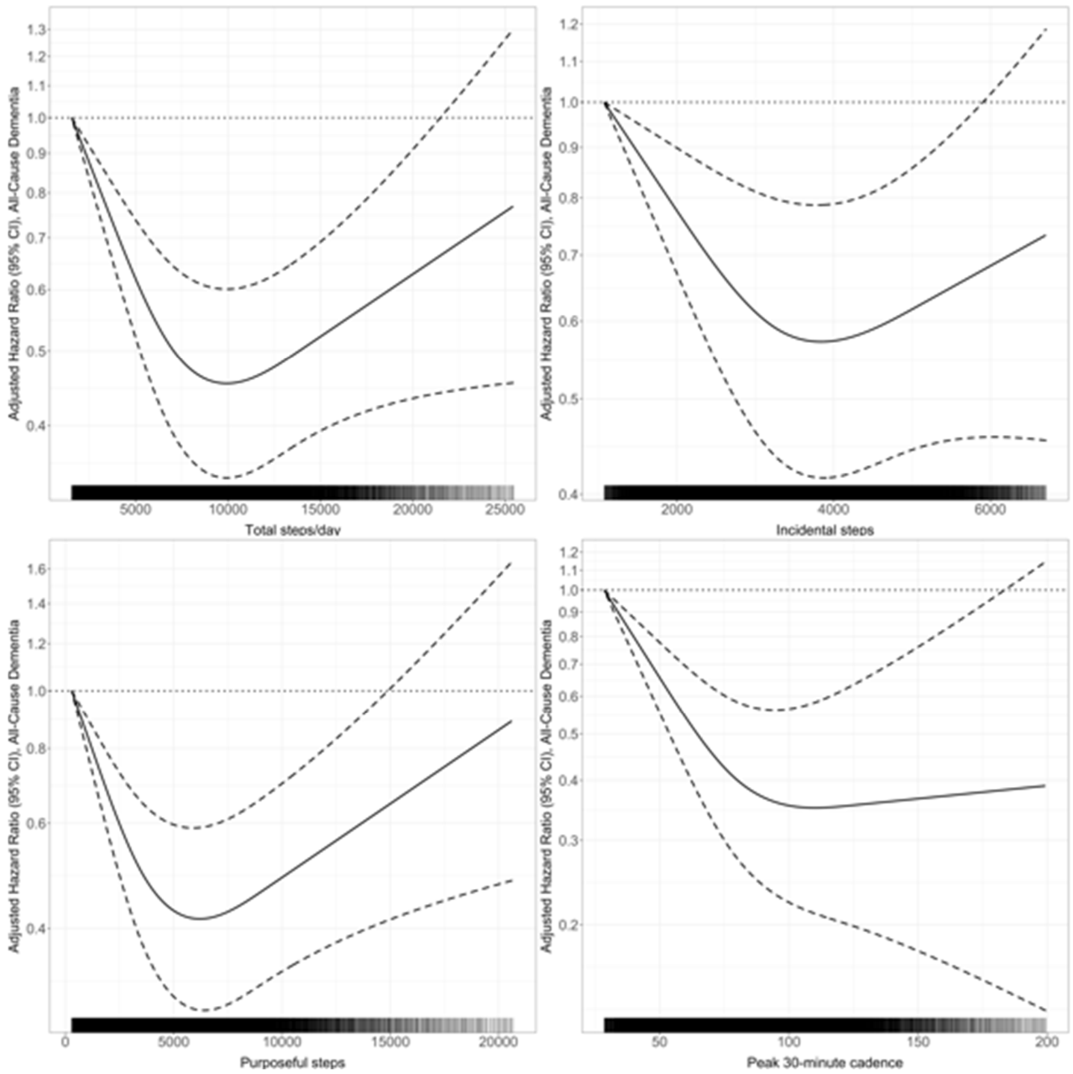

[a]Adjusted for age, sex, ethnicity, education, Townsend deprivation index, smoking, alcohol use, fruit and vegetable consumption, family history of CVD and Cancer, medication use (cholesterol, insulin, and hypertension), accelerometer-measured sleep, wear accelerometer days, cholesterol (HDL, LDL, triglycerides), HbA1c, body mass index, and mean arterial pressure. For incidental steps, models were further adjusted for purposeful steps (and vice versa). For peak 30-min steps models were additionally adjusted for total steps per day. Total steps/day, Average of steps accumulated in a day; Incidental steps, Total daily steps at 1-39 steps/minute; Purposeful steps, Total daily steps at ≥40 steps/minute, Peak 30-min cadence, Average steps/minute recorded for the 30 highest, but not necessarily consecutive, minutes in a day. Dose-response associations were assessed with restricted cubic splines with knots at

© 2022 del Pozo Cruz et al. *JAMA Neurology.*

10th, 50th, and 90th centiles of the distribution of the exposure of interest. Hazard ratios are in logarithmic scale.

© 2022 del Pozo Cruz et al. *JAMA Neurology.*
